# Symptoms and signs did not predict outcome after surgery: a prospective study of 143 patients with idiopathic normal pressure hydrocephalus

**DOI:** 10.1007/s00415-024-12248-w

**Published:** 2024-03-05

**Authors:** Kerstin Andrén, Carsten Wikkelsø, Katarina Laurell, Lena Kollén, Per Hellström, Mats Tullberg

**Affiliations:** 1 Hydrocephalus Research Unit, Institute of Neuroscience and Physiology, Department of Clinical Neuroscience, The Sahlgrenska Academy, University of Gothenburg, Gothenburg, Sweden; 2 Department of Biomedical and Clinical Sciences, Neurobiology, Linköping University, Linköping, Sweden

**Keywords:** Hydrocephalus, Normal pressure hydrocephalus, Cognitive disorders and dementia, Ataxia and gait disorders

## Abstract

**Objective:**

To determine the utility of symptoms, signs, comorbidities and background variables for the prediction of outcome of treatment in iNPH.

**Methods:**

A prospective observational study of consecutively included iNPH patients, who underwent neurological, physiotherapeutic and neuropsychological assessments before and after shunt surgery. The primary outcome measure was the total change on the iNPH scale, and patients were defined as improved postoperatively if they had improved by at least five points on that scale.

**Results:**

143 iNPH patients were included, and 73% of those were improved after surgery. None of the examined symptoms or signs could predict which patients would improve after shunt surgery. A dominant subjective complaint of memory problems at baseline was predictive of non-improvement. The reported comorbidities, duration of symptoms and BMI were the same in improved and non-improved patients. Each of the symptom domains (gait, neuropsychology, balance, and continence) as well as the total iNPH scale score improved significantly (from median 53 to 69, *p* < 0.001). The proportions of patients with shuffling gait, broad-based gait, paratonic rigidity and retropulsion all decreased significantly.

**Discussion:**

This study confirms that the recorded clinical signs, symptoms, and impairments in the adopted clinical tests are characteristic findings in iNPH, based on that most of them improved after shunt surgery. However, our clinical data did not enable predictions of whether patients would respond to shunt surgery, indicating that the phenotype is unrelated to the reversibility of the iNPH state and should mainly support diagnosis. Absence of specific signs should not be used to exclude patients from treatment.

## Introduction

The clinical picture of idiopathic normal pressure hydrocephalus (iNPH) comprises disturbed gait and balance, cognitive impairment and urinary incontinence, treatable by CSF shunting [1]. In modern studies, around 80% of treated patients experience improved symptoms [2]. In spite of extensive research into a wide range of radiological [3, 4], biochemical [5] and hydrodynamic [6–8] markers, to date no specific test can adequately diagnose iNPH or predict which patients will benefit from shunt surgery. Reliable tests for the prediction of outcomes are warranted.

Concerning symptoms and signs, several studies have suggested that either the presence of the complete triad [1] or a clinical picture with a predominant gait disturbance [9], are prognostically favorable. Agerskov et al. showed that the sign of shuffling gait was predictive of improvement post-shunting [1]. Earlier studies have been small or limited with regard to studied symptomatology. The POiNT (Prediction of Outcome in iNPH) study was designed to evaluate the predictive value of clinical, CSF and MRI markers on outcome after shunt surgery, and this paper aims to explore the predictive value of a broad panel of symptoms and signs typical for iNPH using an outcome measure specifically developed for iNPH patients.

## Methods

### Study design and inclusion

**Fig. 1:**
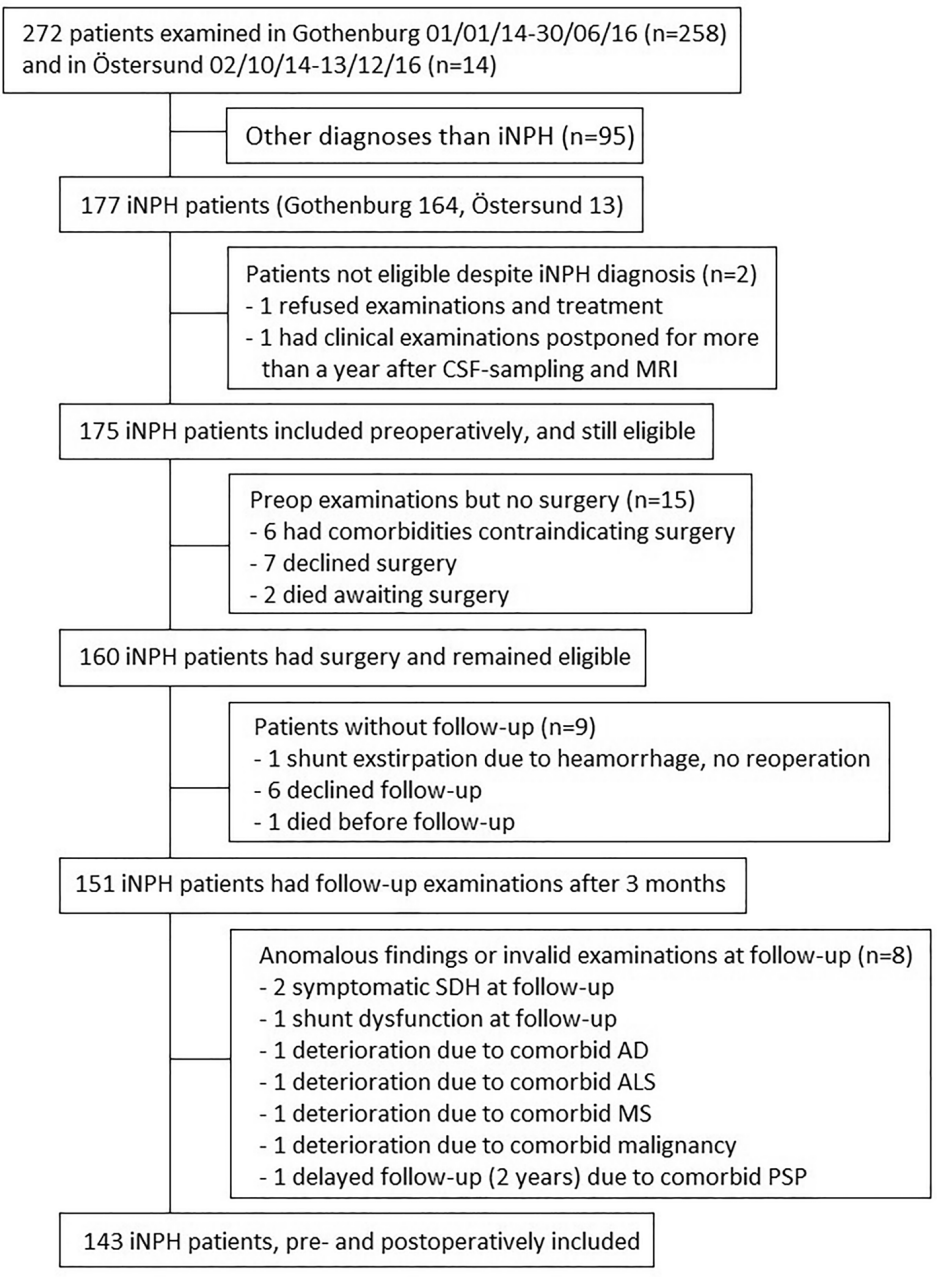
Flow chart of inclusion in the study. *iNPH* idiopathic normal pressure hydrocephalus; *CSF* cerebrospinal fluid; *MRI* magnetic resonance imaging; *SDH* subdural hematoma; *AD* Alzheimer’s disease; *ALS* amyotrophic lateral sclerosis; *MS* multiple sclerosis

The study design was prospective, with consecutive inclusion of patients from two Swedish centers: Sahlgrenska University Hospital in Gothenburg and Östersund Hospital. The inclusion period in Gothenburg was 1 January 2014 to 30 June 2016, and in Östersund 2 October 2014 to 31 December 2016. All 272 patients (Gothenburg *n* = 258 and Östersund *n* = 14) investigated for suspected iNPH in these two centers were assessed for inclusion. A total of 143 patients (Gothenburg, *n* = 130 and Östersund, *n* = 13) were included in the study. Inclusion criterion was a diagnosis of iNPH [5] treated with shunt surgery. Patients who, for different reasons, did not undergo postoperative clinical assessment with the iNPH scale were excluded. A flow-chart is presented in Fig. 1. Demographic and baseline data for the 143 patients are shown in Table 1.

**Table 1:** Baseline demographic and clinical data, with comparisons between improved and non-improved patients

	All patients, *n* = 143	Improved, *n* = 104	Non-improved, *n* = 39	*p*
Demography				
Age, years, mean (SD)	74 (6.7)	74 (6.4)	75 (7.5)	0.327
Sex, female/male, *n* (%)	48/95 (34/66)	36/68 (35/65)	12/27 (31/69)	0.696
BMI, kg/m^2^, mean (SD)	27 (4.0)	27 (3.7)	26 (4.7)	0.134
Modified Rankin Scale (mRS), mdn (IQR)	3 (2–3)	3 (2–3)	3 (2–3)	0.907
mRS score, *n *(%)				
0	0	0	0	
1	5 (3.5)	4 (3.8)	1 (2.6)	
2	57 (40)	40 (39)	17 (44)	
3	59 (41)	46 (44)	13 (33)	
4	18 (13)	11 (11)	7 (18)	
5	4 (2.8)	3 (2.9)	1 (2.6)	
Comorbidities				
Hypertension, *n* (%)	80 (56)	60 (58)	20 (51)	0.572
Diabetes, *n* (%)	42 (29)	33 (32)	9 (23)	0.410
Cardiovascular disease, *n* (%)	44 (31)	31 (30)	13 (33)	0.689
iNPH scale				
Gait, median (IQR)	44 (30–70)	44 (31–67)	44 (29–85)	0.782
Balance, median (IQR)	67 (50–67)	67 (50–67)	67 (50–67)	0.986
Neuropsychology, median (IQR)	48 (33–60)	48 (35–60)	44 (28–63)	0.196
Continence, median (IQR)	60 (40–80)	60 (40–80)	60 (40–80)	0.605
Total score, median (IQR)	53 (41–68)	54 (42–65)	50 (40–76)	0.966
Gait/balance/neuropsychological tests				
TUG time, s, median (IQR)	20 (14–26)	20 (13–26)	20 (14–30)	0.643
TUG steps, *n,* median (IQR)	20 (15–24)	19 (15–23)	22 (16–27)	0.175
Stand up from chair in 30 s, *n*, median (IQR)	6 (3.25–9)	6 (3–8)	6 (4–9)	0.513
Backwards gait 3 m time, s, median (IQR)	13 (8–21)	12 (8–19)	18 (8–22)	0.275
Backwards gait 3 m steps, *n*, median (IQR)	17 (13–23)	16 (13–23)	18 (13–26)	0.595
Steps to turn 180°, *n*, median (IQR)	4 (3.5–6)	4.0 (3.0–6.0)	4.3 (3.5–5.0)	0.871
Romberg’s test (max 60 s), s, median (IQR)	20 (4–60)	19 (4–60)	23 (5–60)	0.853
MMSE, median (IQR)	26 (23–28)	26 (24–28)	26 (22–28)	0.655
Identical forms test, median (IQR)	11.5 (5.9–16)	11.1 (6.0–15.5)	14.4 (6.5–16)	0.424
Bingley memory test, mean (SD)	4 (1.6)	4.0 (1.5)	4.1 (1.8)	0.739
Clinical evaluations and neurological signs				
Leg paratonic rigidity, *n* (%)	99 (69)	72 (69)	27 (69)	1.0
Retropulsion, *n* (%)	68 (52)	49 (52)	19 (53)	1.0
Shuffling gait, *n* (%)	102 (71)	71 (68)	31 (80)	0.218
Freezing of gait, *n *(%)	44 (31)	33 (32)	11 (28)	0.839
Broad-based gait, *n* (%)	106 (74)	77 (74)	29 (74)	1.0
Cerebellar dystaxia, *n* (%)	21 (15)	13 (13)	8 (21)	0.285
Focal neurological signs, *n* (%)	28 (20)	21 (20)	7 (18)	1.0
Sleep, hours per 24 h, mean (SD)	9.2 (2.4)	9.3 (2.5)	8.8 (2.4)	0.289
Number of affected domains (gait/balance/cognitive/urinary), *n* (%)				
1	2 (1.4)	1 (1.0)	1 (2.6)	0.796
2	16 (11)	11 (11)	5 (13)	
3	43 (30)	33 (32)	10 (26)	
4	82 (57)	59 (57)	23 (59)	

### Clinical examinations

All patients underwent clinical examinations by a neurologist, a neuropsychologist, and a physiotherapist, MRI of the brain with standardized sequences, including perfusion protocols, and lumbar puncture for assessment of ICP and routine analyses pre- and in median five months, postoperatively. Ventricular CSF was also sampled perioperatively, and from the Rickham reservoir, postoperatively. Results from the radiological and CSF biochemical investigations will be published separately. Decisions for surgery were made at multidisciplinary conferences. Whenever the diagnosis was considered uncertain, mostly due to indistinct or atypical symptomatology, the CSF tap test or a lumbar infusion test was used to support the diagnosis.

At the baseline examination, the patients’ body mass index (BMI) and any previously diagnosed comorbidity of hypertension, diabetes and cardiovascular disease were registered.

The clinical ratings included the iNPH scale introduced by Hellström [10], where the total score is the mean of four domain scores: gait, balance, cognitive function, and continence. The presence of impaired function within each domain was defined as a gait score ≤ 90, a balance score ≤ 80, a neuropsychology score ≤ 80, and a continence score ≤ 80. Patients were defined as “Improved” if postoperatively their iNPH scale had improved by at least five points, otherwise they were considered “Non-improved” [10]. The threshold of five points for clinically relevant improvement has been used in previous studies [3, 11–14], originally based on clinical experience. The iNPH scale score is derived from a range of standardized quantitative tests and qualitative scales, measured by three different examiners of the three different professions (physician, physiotherapist, neuropsychologist). As the iNPH scale was used as the outcome measure in this study, the tests and scales that are comprised in that scale were not included in the analyses of the clinical variables’ predictive value.

Functional disability was rated with the modified Rankin Scale (mRS) [15] (0–5).

In addition to the ratings and assessments that constitute the iNPH scale, several other observations were included in the pre- and postoperative examinations of the patients. Gait and motor function were assessed by the Timed-up-and-go test [16], the time and number of steps to walk three meters backwards, the number of times the patients were able to stand up from a chair during the 30 s, [17] and the number of steps needed to turn 180°. Balance was measured by the number of seconds achieved in Romberg’s test (maximum 60 s) along with the direction of postural imbalance. Neuropsychological functions were assessed by the Mini-Mental State Examination (MMSE) [18], Identical forms test (test of perceptual speed and accuracy, score 0–60), and Bingley memory test: a task where the patients are asked to memorize pictures of 12 different items—the test is performed twice and the score represents the mean number of items retained (score 0–12) [19]. The number of hours of sleep per 24 h was registered, and, finally, the presence or absence of the following clinical neurological signs were recorded: broad-based or shuffling gait, freezing of gait, paratonic rigidity in the lower extremities, cerebellar ataxia, and focal neurological signs.

All clinical variables were assessed pre- and postoperatively. All examinations were performed within the frame of routine care. The majority of the patients were assessed by the same specialist neuropsychologist and physiotherapist, and consultant or resident neurologists, in the latter case supervised by consultants.

At the baseline evaluation patients and/or their next-of-kin were interviewed about the duration of symptoms perceived as iNPH symptoms. They were also asked which was the first presenting symptom (impaired gait/balance/memory/continence, several simultaneously, headache, or other), and about the dominating subjective complaint at present (impaired gait/balance/memory/continence, headache, or other).

### Treatment

The insertion of ventriculoperitoneal shunts was performed in 139 patients, and ventriculo-atrial shunts in four patients. The choice of operation technique was based on the surgeons’ assessment of individual patients’ prerequisites. PS Medical Strata, Medtronic (Goleta, California, USA) valves with an antisiphon device (ASD) and a standard setting of 1.5 were used in all cases.

All shunts were deemed working at the postoperative assessment. Shunt function was assessed by evaluation of clinical symptoms and CT or MRI. If doubts regarding shunt patency remained following CT or MRI, a radionuclide shuntography [20] or a lumbar infusion test was performed.

### Complications

Before the postoperative follow-up, 12 patients (8.4%) had complications following shunt surgery. Eight patients (5.6%) had major complications requiring new surgery: six had distal mechanical shunt failures (misplacement or obstruction) corrected by surgical revisions, one had a subdural hematoma requiring surgical evacuation and temporary ligation of the shunt, and one had a CNS infection requiring extraction and later reinsertion of a shunt. Four patients (2.8%) had minor complications: two had hygromas requiring upregulation of their shunt valve opening pressure, one had both septicemia related to a urinary catheter placement and gastrointestinal bleeding, prompting termination of antithrombotic treatment, and one had a postoperative transient fever.

### Standard protocol approvals, registrations, and patients consents

The study was performed in accordance with the Declaration of Helsinki and was approved by the Regional Ethical Review Board in Gothenburg (Dnr 328-14, T439-15). All patients or their next of kin gave written consent to inclusion in the study.

### Statistics

For comparisons of ordinal or continuous variables between improved and non-improved patients, the Mann–Whitney *U* test was used. For comparisons between pre- and postoperative data, the Wilcoxon signed-rank test was used for ordered categorical and not normally distributed continuous variables, and the student’s t-test was used for normally distributed continuous variables. Proportions were compared by use of Fisher’s exact test or the Chi-square test.

To assess the predictive value of baseline clinical variables, ROC analyses were performed and odds ratios with 95% confidence intervals were calculated by univariable logistic regression analyses. The dependent variable was an improvement by ≥ 5 points on the iNPH scale (yes/no). Variables with < 5 observations were not tested. Further, correlations between the baseline variables and the delta-iNPH-scale score (i.e., the difference between pre- and postoperative scores) were tested by Pearson’s correlation analysis, and univariable linear regression analyses were performed for variables with a significant correlation with the delta-iNPH-scale score. All significance tests were two-tailed, and alpha was set to 0.05, without corrections for multiple testing. For inclusion in multivariable logistic regression analysis, the significance level was set at *p* < 0.1, but no variable reached this significance level. Analyses were performed with IBM SPSS version 29.

### Data availability

Anonymized data not published within this article will be made available upon request from any qualified investigator.

## Results

At the postoperative examination, in the median five (IQR 4–7) months after surgery, 104 (73%) of patients were improved by at least five points on the iNPH scale. There was no difference in the baseline demographic or clinical variables between the 104 improved and the 39 non-improved patients (Table 1).

Regarding the anamnestic information, the reported symptom duration did not differ between the groups (Table 2). Of the 39 non-improved patients, 26% (*n* = 10) had reported memory impairment as the dominating subjective complaint at the baseline clinical evaluation, compared to 7.8% (*n* = 8) of the improved patients (*p* = 0.009). The percentages of patients reporting gait or balance difficulties as dominating subjective complaint did not differ significantly in improved vs non-improved patients, neither when analyzed separately nor merged (total of 69 + 46 = 115 patients, *p* = 0.154). A report of headache as a presenting symptom was seen in 3 patients, all subsequently non-improved by shunt surgery; apart from that, no other debut symptom was over-represented in improved or non-improved patients (Table 2).

**Table 2:** Anamnestic data at baseline clinical evaluation, with comparisons between improved and non-improved patients

	All patients, *n* = 143	Improved, *n* = 104	Non-improved, *n* = 39	*p*
Duration of symptoms, mts, mdn (IQR)	36 (21–48)	33 (19–60)	36 (24–48)	0.878
First presenting symptom, *n* (%)				
Gait impairment	56 (39)	39 (38)	17 (44)	0.566
Balance impairment	37 (26)	30 (29)	7 (18)	0.206
Memory impairment	16 (11)	12 (12)	4 (10)	1.0
Incontinence	4 (2.8)	3 (2.9)	1 (2.6)	1.0
Several simultaneously	25 (18)	19 (18)	6 (15)	0.807
Headache	3 (2.1)	0	3 (7.7)	0.019
Other	2 (1.4)	1 (1.0)	1 (2.6)	0.472
Dominating subjective complaint, *n *(%)				
Gait impairment	69 (48)	52 (51)	17 (44)	0.574
Balance impairment	46 (32)	35 (34)	11 (28)	0.688
Memory impairment	18 (13)	8 (7.8)	10 (26)	0.009
Incontinence	4 (2.8)	3 (2.9)	1 (2.6)	1.0
Headache	0	0	0	
Other	5 (3.5)	5 (4.9)	0	0.323

There were postoperative significant improvements in each of the domains of the iNPH scale as well as in the total iNPH scale score, with a median increment from 53 to 69 points (*p* < 0.001) (Table 3). The modified Rankin Scale improved from a median 3 to 2 (IQR 2–3 both pre- and postoperatively, *p* < 0.001), and 36% (*n* = 48) had improved by at least one scale score. Improvement was seen in all registered symptoms and signs except for MMSE, Bingley’s memory test, freezing of gait, cerebellar dystaxia, and focal neurological signs (Table 3). The number of hours of sleep per 24 h diminished from a mean of 9.2 to 8 (*p* < 0.001). Results in the Identical forms test were improved. Half as many patients had retropulsion or shuffling gait after surgery compared to before. The median number of seconds managed in Romberg’s test increased from 20 to 60 (*p* < 0.001) and the median number of steps to turn 180° was improved from four to three. Further, the proportions of patients with paratonic rigidity in the legs and with broad-based gait were smaller, postoperatively. The number of seconds and steps needed in the TUG test as well as in the 3 m backwards walk test decreased (< 0.001 for all). The median number of times patients were able to stand up from a seated position in 30 s increased from six to eight (*p* < 0.001) (Table 3).

**Table 3:** Results from pre- and postoperative examinations in 143 iNPH patients

	Preoperative	Postoperative	*p*
Modified Rankin Scale (mRS), median (IQR)	3 (2–3)	2 (2–3)	< 0.001
mRS score, *n *(%)			
0	0	1 (0.7)	
1	5 (3.5)	20 (14)	
2	57 (39.9)	66 (46.2)	
3	59 (41.3)	45 (31.5)	
4	18 (12.6)	10 (7)	
5	4 (2.8)	1 (0.7)	
iNPH scale			
Gait, median (IQR)	44 (30–70)	67 (43–92)	< 0.001
Balance, median (IQR)	67 (50–67)	67 (67–83)	< 0.001
Neuropsychology, median (IQR)	48 (33–60)	55 (38–73)	< 0.001
Continence, median (IQR)	60 (40–80)	80 (60–100)	< 0.001
Total score, median (IQR)	53 (41–68)	69 (53–81)	< 0.001
Gait/balance/neuropsychological tests			
TUG time, s, median (IQR)	20 (14–26)	14 (10–19)	< 0.001
TUG steps, *n*, median (IQR)	20 (15–24)	15 (13–19)	< 0.001
Stand up from chair in 30 s, *n*, median (IQR)	6 (3.25–9)	8 (5–11)	< 0.001
Backwards gait 3 m time, s, median (IQR)	13 (8–21)	8 (5–12)	< 0.001
Backwards gait 3 m steps, *n*, median (IQR)	17 (13–23)	11 (8–15)	< 0.001
Steps to turn 180°, *n*, median (IQR)	4 (3.5–6)	3 (2–4)	< 0.001
Romberg’s test (max 60 s), s, median (IQR)	20 (4–60)	60 (10–60)	< 0.001
MMSE, median (IQR)	26 (23–28)	26 (23–28)	0.576
Identical forms test, median (IQR)	11.5 (5.9–16)	13 (8–19)	< 0.001
Bingley memory test, mean (SD)	4 (1.6)	4.3 (1.9)	0.075
Clinical evaluations and neurological signs			
Leg paratonic rigidity, *n* (%)	99 (69)	83 (62)	< 0.001
Retropulsion, *n* (%)	68 (52)	34 (35)	< 0.001
Shuffling gait, *n* (%)	102 (71)	53 (38)	< 0.001
Freezing of gait, *n* (%)	44 (31)	15 (11)	0.237
Broad-based gait, *n* (%)	106 (74)	78 (55)	0.020
Cerebellar dystaxia, *n* (%)	21 (15)	14 (11)	1.0
Focal neurological signs, *n* (%)	28 (20)	18 (14)	0.219
Sleep, h/24 h, mean (SD)	9.2 (2.4)	8 (1.9)	< 0.001

None of the baseline demographic or clinical recordings were found to predict beneficial post-surgical outcome in univariable logistic regression analyses with a significance level of < 0.10 (Table 4). Of the anamnestic information, a report of dominant subjective complaint of memory problems at baseline was significantly predictive of non-improvement (Table 4), remaining significant when adjusted for age and total iNPH scale score; adjusted OR 0.26, 95% CI 0.09–0.68, *p* = 0.007. The ROC analyses yielded AUC values close to 0.5 for all variables, meaning that the studied clinical variables’ predictive value was low (Table 4).

**Table 4:** The demographic, clinical and anamnestic factors’ predictive value for postoperative improvement by at least five points on the iNPH scale, univariable logistic regression analyses and ROC analyses

	OR	95% CI	*p*	AUC (95% CI)
Demography				
Age, years	0.97	0.92–1.03	0.33	0.41 (0.30–0.51)
Sex, female/male	1.19	0.54–2.63	0.67	0.48 (0.38–0.59)
BMI, kg/m^2^	1.08	0.98–1.19	0.14	0.60 (0.49–0.71)
Modified Rankin Scale, 1–2 vs 3–5	0.86	0.41–1.79	0.68	0.52 (0.41–0.63)
Comorbidities				
Hypertension, y/n	0.77	0.37–1.62	0.49	0.53 (0.43–0.64)
Diabetes, y/n	0.65	0.28–1.51	0.31	0.54 (0.44–0.65)
Cardiovascular disease, y/n	1.18	0.54–2.59	0.68	0.48 (0.38–0.59)
Clinical evaluations and neurological signs				
MMSE score	1.01	0.93–1.11	0.76	0.52 (0.42–0.63)
Identical forms test, score	0.98	0.93–1.04	0.45	0.46 (0.34–0.57)
Bingley memory test, score	0.96	0.75–1.22	0.74	0.52 (0.40–0.64)
Sleep, h/24 h	1.09	0.93–1.29	0.29	0.57 (0.46–0.68)
Leg paratonic rigidity, y/n	1.00	0.45–2.22	1.0	0.50 (0.39–0.61)
Retropulsion, y/n	1.03	0.48–2.22	0.95	0.50 (0.39–0.61)
Shuffling gait, y/n	1.8	0.75–4.34	0.19	0.44 (0.34–0.55)
Freezing of gait, y/n	0.85	0.38–1.90	0.68	0.52 (0.41–0.62)
Broad-based gait, y/n	1.02	0.44–2.36	0.97	0.50 (0.39–0.61)
Cerebellar dystaxia, y/n	1.85	0.70–4.88	0.22	0.46 (0.35–0.57)
Focal neurological signs, y/n	0.89	0.35–2.31	0.81	0.51 (0.40–0.62)
Gait/balance tests				
TUG time, s	0.99	0.97–1.02	0.65	0.47 (0.36–0.59)
TUG steps, *n*	0.98	0.94–1.01	0.19	0.42 (0.31–0.54)
Stand up from chair in 30 s, *n*	0.94	0.84–1.05	0.27	0.46 (0.33–0.59)
Backwards gait 3 m time, s	0.99	0.97–1.02	0.63	0.44 (0.31–0.56)
Backwards gait 3 m steps, *n*	1.00	0.97–1.04	0.86	0.47 (0.35–0.59)
Steps to turn 180°, *n*	1.01	0.87–1.17	0.95	0.51 (0.40–0.62)
Romberg’s test (max 60 s), s	1.00	0.99–1.01	0.95	0.49 (0.39–0.59)
Anamnestic data				
Duration of symptoms, mts	1.00	0.99–1.02	0.69	0.51 (0.40–0.61)
First symptom: gait, y/n	0.78	0.34–1.64	0.51	0.47 (0.36–0.58)
First symptom: balance, y/n	1.85	0.74–4.66	0.19	0.55 (0.45–0.66)
First symptom: memory, y/n	1.14	0.35–3.78	0.83	0.51 (0.40–0.61)
First symptom: several, y/n	1.23	0.45–3.35	0.69	0.51 (0.41–0.62)
Dominating complaint: gait, y/n	1.32	0.63–2.77	0.45	0.53 (0.43–0.64)
Dominating complaint: balance, y/n	1.31	0.58–2.94	0.51	0.53 (0.42–0.63)
Dominating complaint: memory, y/n	0.24	0.09–0.68	0.007	0.41 (0.30–0.52)

*OR* Odds ratio; *CI* confidence interval; *AUC* area under the curve; *BMI* body mass index; *MMSE* mini-mental state examination; *TUG* timed up-and-go test; *y/n* yes/no

A few significant associations were noticed but they were all weak. Older patients improved less than younger in univariable linear regression analysis: *R* = − 0.18, *p* = 0.031, *β* = 0.3. The number of affected domains (1–4) did correlate with improvement: *R* = 0.19, *p* = 0.021, *β* = 2.8. Finally, the presence of impairment in all four symptom domains correlated with improvement: *R* = 0.21, *p* = 0.014, *β* = 4.8. None of the other baseline variables correlated significantly with the change in total iNPH scale score.

There was no difference in improvement rates between patients with or without any complication before the postoperative investigation (*p* = 1.0). Neither did improvement rates differ for patients with minor (*p* = 0.30), nor major (*p* = 0.45) complications compared to those without.

## Discussion

Here, we made a substantial effort to identify clinical variables of predictive value. Our study included well over 100 patients, it was prospective and consecutive, and involved the registration of around 30 different symptoms and signs, comorbidities and BMI. To the best of our knowledge, this is the largest study of its kind so far addressing this question.

It has been a major challenge for previous research to predict outcomes after shunt treatment in NPH and to identify the approximate 20% of patients not improved by shunt surgery [2, 11]. Available CSF dynamic or drainage tests all show low negative predictive values [7, 8] and the use of CSF and MRI biomarkers have, so far, not improved the identification of non-responders [3, 4, 21–23], which is why better predictive markers are highly warranted. Since the diagnosis of iNPH is based on recognition of the typical clinical syndrome in combination with radiological markers, the use of symptoms and signs for prediction is both practical and appealing.

In our sample of diagnosed and operated iNPH patients, none of the preoperative clinical tests could aid in telling apart patients who later benefitted from shunt surgery from those who did not: comparisons of clinical baseline data between improved and non-improved patients revealed no preoperative differences. The odds ratios for improvement were non-significant for all those baseline variables. Only the anamnestic information of memory impairment being the dominating subjective complaint, was found to have a predictive value, with a lower improvement rate of 44% in that group of 18 patients, compared to 73% in the whole cohort. These 18 patients do not stand out from the remaining cohort of 125 patients with regard to clinical baseline variables: their age was similar, *p* = 0.58, and they had a median MMSE of 25; IQR 20–27, without significant difference from the remaining cohort, *p* = 0.21. Neither was any significant difference seen in their preoperative total iNPH scale score, *p* = 0.54, nor in the neuropsychology or gait domain score of the iNPH scale, *p* = 0.11 and *p* = 0.31. Impairment was seen in all four symptom domains of the iNPH scale at baseline in ten of those 18 patients, three domains in four, two domains in three patients, and in one impairment was seen only in the neuropsychology domain.

Three of the patients (2%) reported headache as the presenting symptom—none of them later improved after shunt surgery. A possible interpretation of this finding would be that headache is not a typical presenting symptom of shunt-responsive iNPH. But, the reliability of the finding is low: interviewing patients about what was the presenting symptom generally yields uncertain answers.

Weak, yet statistically significant, correlations were found between the degree of improvement and age and with the number of affected symptom domains. The postoperative change in the iNPH scale score would be 0.3 points lower per additional year of age in a univariable linear regression model. Higher age as a negative predictive factor has been reported previously [24, 25], whereas others did not find less beneficial results in older patients [1, 26, 27]. Considering the high rates of improvement also in older patients, and the weak correlation with age reported here, our opinion is that age should not be a determinant factor in the decision making of shunting a patient.

The finding that the more complete the clinical syndrome the better the outcome, as well as the positive prognostic value of the presence of the full tetrad, are interesting results that corroborate a previous study from our group [1]. These findings are reasonable given the potential reversibility of all symptoms in iNPH—the more symptoms that can be reversed, the better the improvement—and also gives support to the notion that the mechanism generating the iNPH symptom in some way is related to reversibility. The results also add value in the clinical setting: patients with more paramount symptoms should be considered eligible for surgery. However, all these significant associations were weak, adding no substantial support to the identification of possible responders or non-responders in the clinical routine. None of the other clinical factors could predict how much a patient would improve. Neither was the presence of diabetes, cardiovascular disease nor the patients’ BMI associated with the outcome. Although an increased burden of cerebrovascular disease and vascular risk factors are described in patients with iNPH [28–32], the impact on outcome after shunt surgery seems to be limited [11, 33–37]. In a previous study of 979 iNPH patients from the Swedish Hydrocephalus Quality Registry, we found neither cardiovascular nor cerebrovascular disease to have a negative influence on improvement within five years after shunt surgery [38]. In line with our findings, Klinge et al. recently reported no association between comorbidity burden and outcome in 208 iNPH patients after shunt surgery [39].

Notably, in the present study, symptom duration was not associated with outcome, which might imply that iNPH does not progress into an irreversible state. On the other hand, disease duration is difficult to ascertain, which could explain why the well-documented negative effect of time on prognosis was not identified in this study [40].

As none of our findings are really of any help in identifying possible responders or non-responders, clinical measures should probably be used mainly for diagnostics and not for prediction of outcome. Another reasonable conclusion of this work is that patients lacking the full panel of typical iNPH symptoms should also be considered for shunt surgery as should patients with a heavy symptom burden, since the symptom severity did not predict whether the patients improved or not.

One possible reason for the lack of strong predictive associations is that factors not assessed here are related to reversibility and improvement. We only included data on the comorbidity of hypertension, diabetes, and cardiovascular disease, whereas e.g., Alzheimer’s disease, parkinsonian disorders or orthopedic conditions such as hip, knee or spinal arthrosis—all with potential influence on outcome—were not included. However, patients were selected for surgery based on iNPH typical symptoms and the absence of signs of comorbidities thought to explain symptomatology or affect outcome in a significant way. As accounted for in Fig. 1, we also chose to exclude patients after the postoperative follow-up if they had progressed substantially in comorbid conditions fogging the assessment of whether the iNPH symptoms had improved or not. However, the remaining included patients are also older people with a range of comorbidities that affect the performance level in the different outcome measures—an inherent confounder in all studies on iNPH patients. Future studies should include a more detailed assessment of the prognostic value of a broader range of comorbidities [41].

Further, no detailed assessment of radiological changes or biochemical markers was included here: all patients fulfilled radiological criteria for iNPH and had no significant evidence of other neurodegenerative disorders in the CSF. We plan to explore the predictive value of MR perfusion and diffusion, as well as of CSF biomarkers, in the same patient sample in future publications.

Another possible factor related to the lack of improvement could be a suboptimal shunt function. We used Medtronic Strata programmable valves with an ASD and a standard setting of 1.5. In our clinical routine, if a patient is not improved, shunt function is tested and if the shunt is found working, reduction of the opening pressure is considered, weighing patients’ individual risk vs benefit. We cannot exclude that patients in the unimproved group would have improved if the opening pressure had been further reduced. However, there is good evidence that lowering the opening pressure below 12 cm H2O does not improve symptoms and signs but heightens the risk of complications [42–44]. The pressure setting in this study is substantially lower than 12 cm H2O in the supine position, which is why we assume that it is probably not the pressure setting that explains why some are non-responders. Volumetric imaging studies have shown positive correlations between postoperative improvement and reduction of ventricular volume [45], pointing at shunt CSF flow, shunt-induced ICP reduction, and brain elasticity or stiffness as factors that can be involved in reversibility and outcome.

The response to shunt surgery in this patient sample needs to be commented: 104 patients improved at least 5 points on the iNPH scale and another 27 showed a postoperative change of 0–4 points, i.e. 92% (131/142) were either improved or showed no further decline in their symptoms according to the chosen cutoff. In light of recent reports on AD treatment aimed at slowing down disease progression with e.g. lecanemab, where researchers describe findings of a 64% probability of active treatment being better than placebo [46], one is inclined to consider shunt surgery for iNPH a remarkable treatment.

Symptoms and signs were measured in detail pre- and postoperatively. All the gait and balance measurements improved, as did one of the applied cognitive tests not included in the iNPH scale, the modified Rankin scale, and the number of hours needed to sleep. The proportions of patients with abnormal neurological signs were smaller postoperatively, e.g., broad-based or shuffling gait, freezing of gait, paratonic rigidity in the lower extremities and retropulsion in Romberg’s test. This supports the view that the profile of symptoms and signs in iNPH is unique and clinically different from other degenerative disorders such as Alzheimer’s disease, Parkinson’s disease, and atypical parkinsonian disorders [1]. The high degree of reversibility and the uniformity of the improvement pattern render these clinical measures important when considering a diagnosis of iNPH.

We believe that the merits of this study are considerable and that it is important to conceptualize and incorporate the “negative” result into the understanding of NPH pathophysiology. We have not identified a reversibility factor in the constellation of symptoms and signs, nor in its severity. In some patients, symptoms and signs for some reason become irreversible. Contrary to what could be expected, this irreversibility is not related to the duration of the disease, nor to the severity of its symptoms. Neither is there any support for a biochemical functional limit; CSF biomarker studies have not been able to identify specific levels of impaired or altered metabolism capable of telling responders and non-responders apart, or of predicting the magnitude of improvement [5, 47, 48]. What is more confusing is the fact that improvement in iNPH is associated with a normalization of the pathological CSF biomarker profile; an increase in amyloid precursor proteins [21]. There is a bulk of evidence in support of a metabolic disturbance in predominantly periventricular regions of the brain, including pons, mesencephalon, thalamus and basal ganglia, and frontal periventricular white matter in iNPH related to the CSF dynamic disturbance [21, 47]. It is reasonable to assume that functional disturbances on a cellular level in these regions, including neuronal dysfunction, determine reversibility. These changes present clinically with the typical iNPH phenotype with characteristic symptoms and signs. Over time, the functional disturbance may increase, causing a gradual clinical deterioration, still potentially reversible. Around 20% of iNPH patients do not respond to shunt treatment [2], indicating that, for some reason or at some point, the disturbance becomes less reversible and is no longer influenced by normalization of CSF dynamics. This is probably what happens during the natural course of disease if left untreated, with developing irreversible damage to the brain [40]. The search for markers of this process needs to be pursued. Several studies have pointed at the brain stem region as a generator of typical symptoms in iNPH [49–52]. One of these studies investigated wakefulness in relation to cerebral blood flow in periventricular areas including the mesencephalon, finding an increase in blood flow in patients who no longer had impaired wakefulness post-shunt surgery [50], theoretically connected to the finding in the present paper that the number of hours asleep decreases. Is it possible that there is a primary symptom generator located at the brain stem level that influences the overall degree of reversibility? We believe that future studies of biochemical and metabolic function should focus on these brain stem regions. Here, we are measuring secondary systems, not the primary symptom generator.

The strengths of this study are its prospective design with consecutive inclusion of patients, the large patient sample, including a considerably large group of unimproved patients, the meticulous assessment of clinical symptoms and signs, and the use of an outcome measure developed for iNPH. The inclusion criteria, the proportion of improved patients, and the profile of improvement reported here are the same as in contemporary studies, supporting the representativeness of our study.

The limitations, some of which have been mentioned earlier, consist of our lack of data for better evaluating the influence of comorbidities, the lack of a systematic routine for adjusting shunt opening pressure to optimal clinical performance, and the number of dropouts and exclusions from postoperative follow-up. As patients were examined within the frame of routine care, blinding to whether the patients had had surgery or not was not possible, and the inter-rater reliability was not measured. Another limitation is that the used traditional statistical methods are not able to evaluate potential predictive values of combinations of variables, such as a machine learning approach might have enabled.

As with morphological radiological markers, there is a potential circular reasoning in studying the predictive value of clinical characteristics and symptoms, that are in fact required for the diagnosis of the disease. Further, the symptoms are what the treatment aims to improve, consequently they are what we measure to evaluate the result of the treatment. To do this as accurately as possible, the clinical tests used in the iNPH scale were only used to measure the outcome, and a wide range of other clinical variables were tested for predictive potential.

Finally, a more liberal approach to shunting a wider range of patients might have enabled clearer results on prognostic factors; however, all decisions were made as part of our general clinical routine, and we do not propose that the indications for shunt surgery should be widened for this purpose.

## Conclusion

This study confirms that the recorded clinical signs, symptoms, and impairments in the adopted clinical tests are characteristic findings in iNPH, based on that most of them improved after shunt surgery. However, our clinical data did not enable predictions of whether or not patients would respond to shunt surgery, indicating that the phenotype is unrelated to the reversibility of the iNPH state and should mainly support the diagnosis. Absence of specific signs should not be used to exclude patients from treatment.
